# Complex neuroanatomy in the rostrum of the Isle of Wight theropod *Neovenator salerii*

**DOI:** 10.1038/s41598-017-03671-3

**Published:** 2017-06-16

**Authors:** Chris Tijani Barker, Darren Naish, Elis Newham, Orestis L. Katsamenis, Gareth Dyke

**Affiliations:** 1 Ocean and Earth Science, National Oceanography Centre, University of Southampton, European Way, Southampton, SO13 3ZH UK; 2 Faculty of Engineering and the Environment, University of Southampton, SO17 1BJ Southampton, UK; 3 µVIS X-ray Imaging Centre, Faculty of Engineering and the Environment, University of Southampton, SO17 1BJ Southampton, UK; 4 Department of Evolutionary Zoology and Human Biology, University of Debrecen, 4032 Debrecen, Egyetem tér 1, Hungary; 5 Center for Interdisciplinary Biosciences, Faculty of Science, Pavol Jozef Safarik University, Jesenna 5, SK-04154 Kosice, Slovakia

## Abstract

The discovery of large, complex, internal canals within the rostra of fossil reptiles has been linked with an enhanced tactile function utilised in an aquatic context, so far in pliosaurids, the Cretaceous theropod *Spinosaurus*, and the related spinosaurid *Baryonyx*. Here, we report the presence of a complex network of large, laterally situated, anastomosing channels, discovered via micro-focus computed tomography (μCT), in the premaxilla and maxilla of *Neovenator*, a mid-sized allosauroid theropod from the Early Cretaceous of the UK. We identify these channels as neurovascular canals, that include parts of the trigeminal nerve; many branches of this complex terminate on the external surfaces of the premaxilla and maxilla where they are associated with foramina. *Neovenator* is universally regarded as a ‘typical’ terrestrial, predatory theropod, and there are no indications that it was aquatic, amphibious, or unusual with respect to the ecology or behaviour predicted for allosauroids. Accordingly, we propose that enlarged neurovascular facial canals shouldn’t be used to exclusively support a model of aquatic foraging in theropods and argue instead that an enhanced degree of facial sensitivity may have been linked with any number of alternative behavioural adaptations, among them defleshing behaviour, nest selection/maintenance or social interaction.

## Introduction

*Neovenator salerii* is an allosauroid theropod from the Wessex Formation of the Isle of Wight, UK (Barremian, Early Cretaceous)^1, 2^, known from a partial skeleton (Museum of Isle of Wight Geology (MIWG) 6348) that comprises extensive cranial, axial, and appendicular material preserved in excellent three-dimensional condition. *Neovenator* is a member of the tetanuran clade Allosauroidea, as evidenced by a suite of cranial and postcranial characters^2–5^. Within Allosauroidea, recent work identifies it a basal member of Neovenatoridae^6^.

A deep, laterally compressed (oreinirostral) skull, large, ziphodont teeth, and a facial and mandibular skeleton considered typical for large, predatory theropods indicate that *Neovenator* was a terrestrial predator that attacked and dispatched vertebrate prey in a manner typical for allosauroids. *Neovenator* has been assumed to have been an apex terrestrial predator, like its carcharodontosaurian close relatives^7^. Analysis of the feeding dynamics of the closely-related *Allosaurus fragilis* indicate powerful ventroflexive angular acceleration of the skull, coupled with minimal shake feeding^8^. Minimal tooth-to-bone contact and the targeting of smaller/juvenile individuals are also predicted^9^. There are no indications that *Neovenator* was unusual in ecology or prey choice with respect to other allosauroids and it is assumed to have been a predator of ornithopods and other mid-sized dinosaurs, a hypothesis supported by bite marks on an associated specimen of the iguanodontian *Mantellisaurus atherfieldensis*.

The osteology of *Neovenator* is well-understood^2^. However, we were intrigued by enlarged foramina present on both the lateral surface of the premaxilla, occasionally set within shallow grooves^2^, and on the anterior ramus of the maxilla and hypothesised that they might be indicative of a rostral neuroanatomy similar to that reported in spinosaurids^10^, and more recently, tyrannosaurids^11^. Complex neuroanatomical systems present in the rostra of many vertebrates have been associated with a function in prey detection. However, poor preservation typically means that they are not sufficiently well-preserved to allow detailed examination^12^. Mini-focus medical CT imaging of the Cretaceous spinosaurid *Spinosaurus aegyptiacus* was previously employed to investigate the internal structure of the enlarged foramina on the surface of its premaxilla. The exceptional volume of these neuroanatomical structures has been linked to an aquatic tactile ability, consistent with narial position, jaw and tooth shape, limb proportions, and reduced medullary cavities in the long bones which have been regarded as indicative of an amphibious or aquatic mode-of-life^10^. These rostral structures of *Spinosaurus* were thus regarded as analogous to the dermal pressure receptors (DPR)^10^ present in crocodylians.

Here, we revisit the cranial morphology of the Isle of Wight theropod *Neovenator* using microfocus μCT to investigate the distribution of its rostral foramina and any internal preservation. We use these data to inform inferences about the palaeobiology and behaviour of *Neovenator* and other large tetanuran theropods, whilst standardising our results to facilitate further analysis of theropod/dinosaurian cephalic vasculature.

## Results

**Figure 1:**
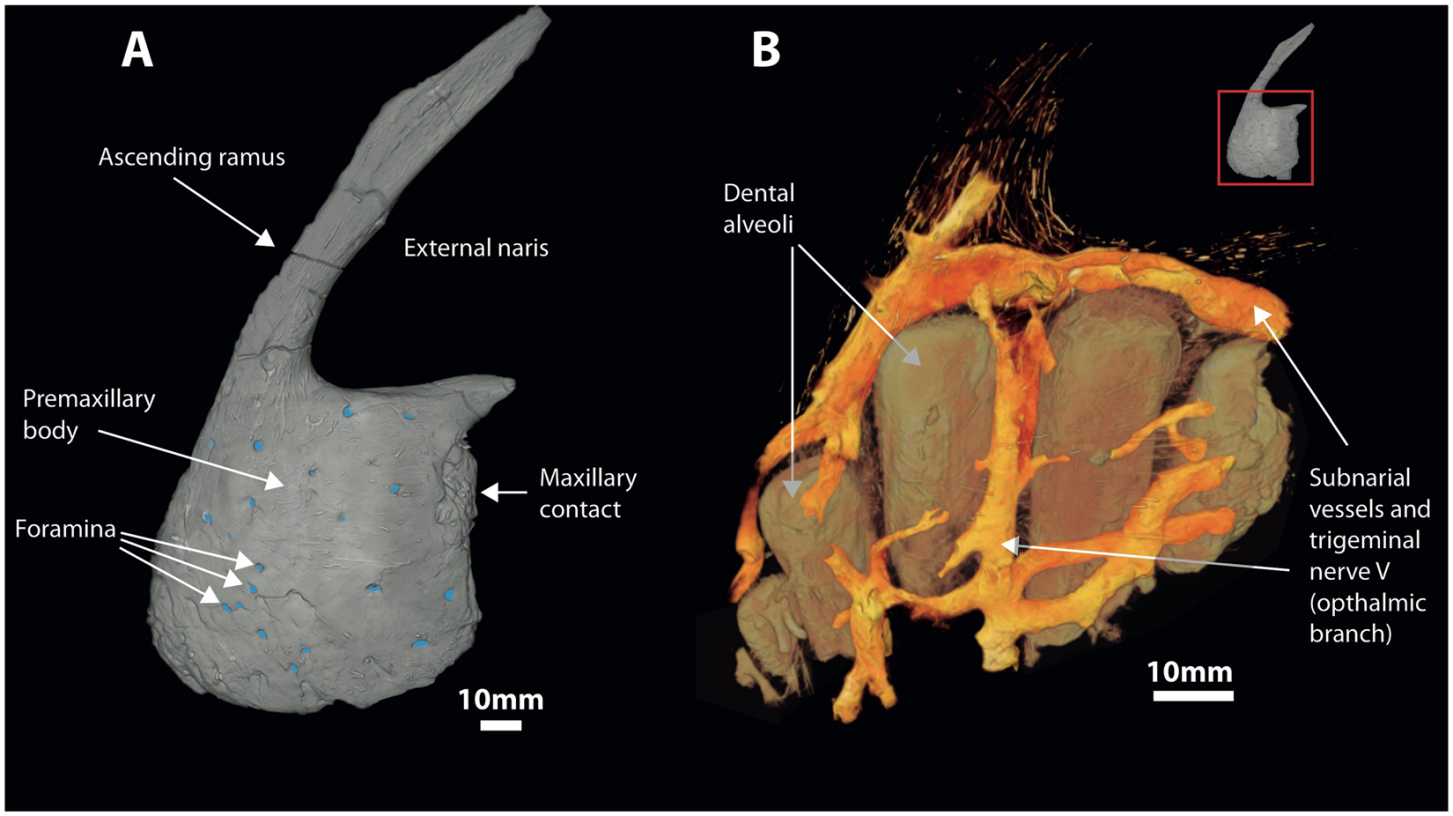
Complex anastomosing neurovasculature surrounding infilled dental alveoli of the premaxilla of *Neovenator*. (**A**) Volume rendering of left premaxilla in lateral view with foramina highlighted (blue). (**B**) Volume rendering of infilled voids.

**Figure 2:**
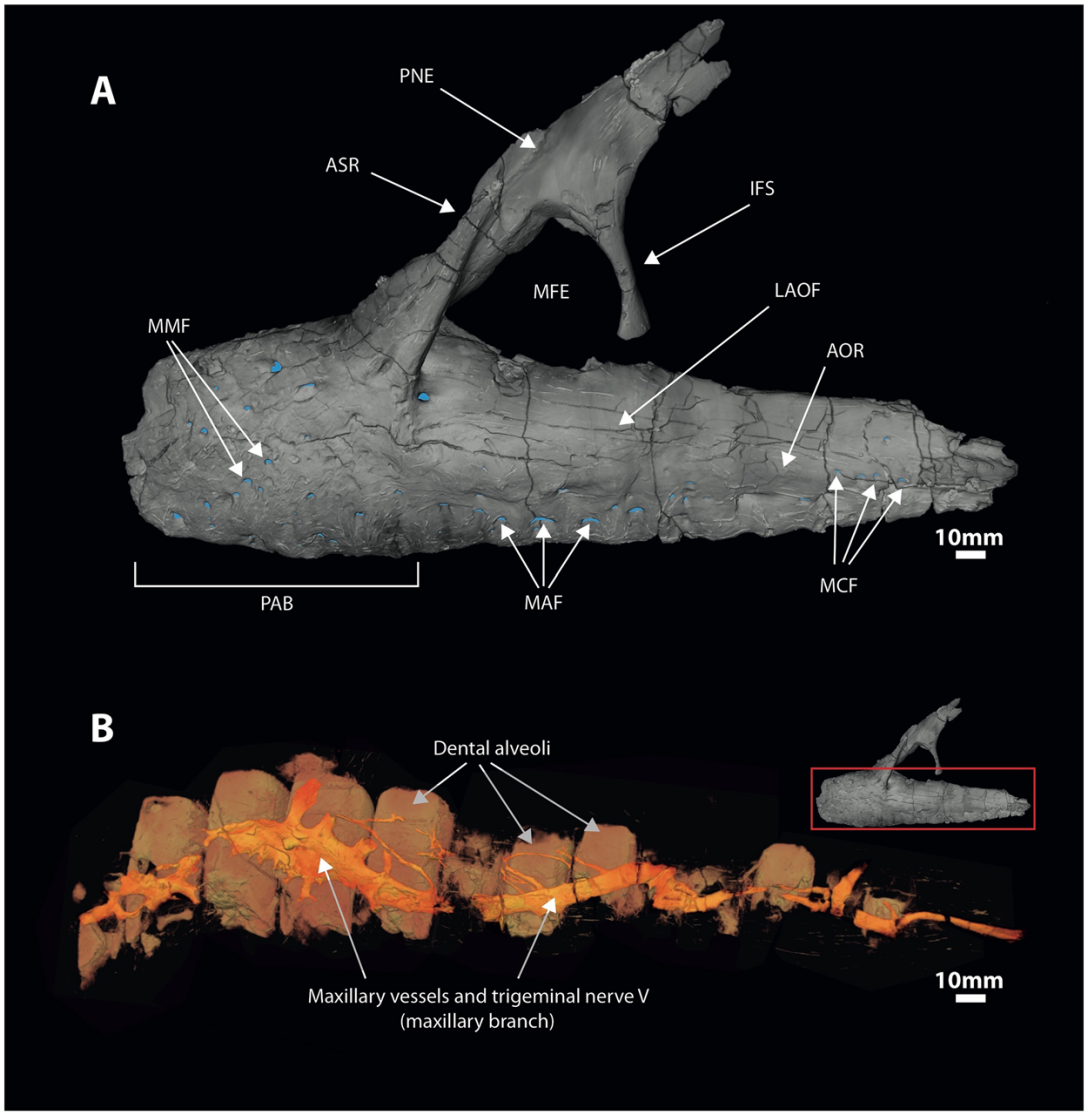
Complex anastomosing neurovasculature surrounding infilled dental alveoli of the maxilla of *Neovenator*. (**A**) Volume rendering of left maxilla in lateral view with foramina highlighted (blue). (**B**) Volume rendering of infilled voids. Abbrevations: **aor**: antorbital ridge; **asr**: ascending ramus; **ifs**: interfenestral strut; **laof**: lateral antorbital fossa; **maf**: maxillary alveolar foramina; **mcf**: maxillary circumfenestra foramina; **mfe**: maxillary fenestra; **mmf**: medial maxillary foramina; **pab**: preantorbital body; **pne**: pneumatic excavation.

**Figure 3:**
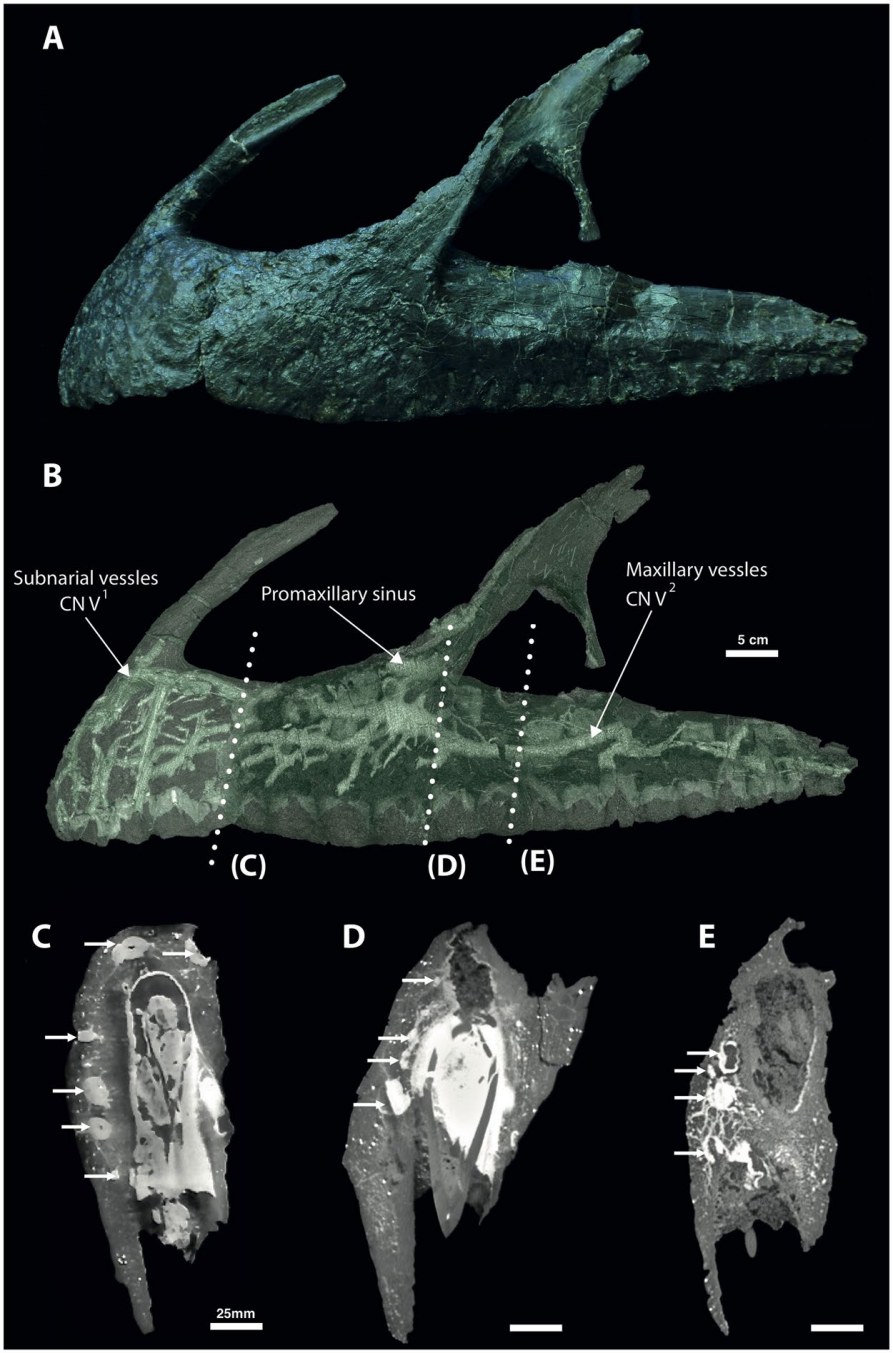
(**A**) Articulated premaxilla and maxilla of *Neovenator* holotype MIWG 6348 in left lateral view (Credit: Roger Benson). (**B**) Volume rendering of the segmented neurovascular network overlaid on the articulated premaxilla and maxilla. (**C**–**E**) μCT virtual sections showing lateral placement of the neurovasculature (white arrows).

Of the material scanned, the left premaxilla and maxilla revealed the best internal preservation. A complex network of canals branch extensively in both elements (Figs 1, 2 and 3); these canals are located lateral to the dental alveoli (they are absent from the medial side), terminating on the external surfaces of the bones (Fig. 3, I-III). In both bones, several canals exit via foramina on the bone surface (Figs 1A and 2B), with the premaxilla and preantorbital body presenting the most (Table 1).

**Table 1:** Foramina count, neurovascular volume, bone volume, and ratio (%) in the premaxilla and maxilla of *Neovenator*.

Element	Bone Anatomy	Foramina count	Nerve volume (cm^3^)	Bone volume (cm^3^)	Ratio
Premaxilla	Premaxillary body	47	1.1	15.0	7.3
Maxilla	Preantorbital body	40	9.2	164.0	5.6
	Anterior body	61	11.7	261.3	4.5
	Jugal ramus	17	1.5	67.8	2.2

**Figure 4:**
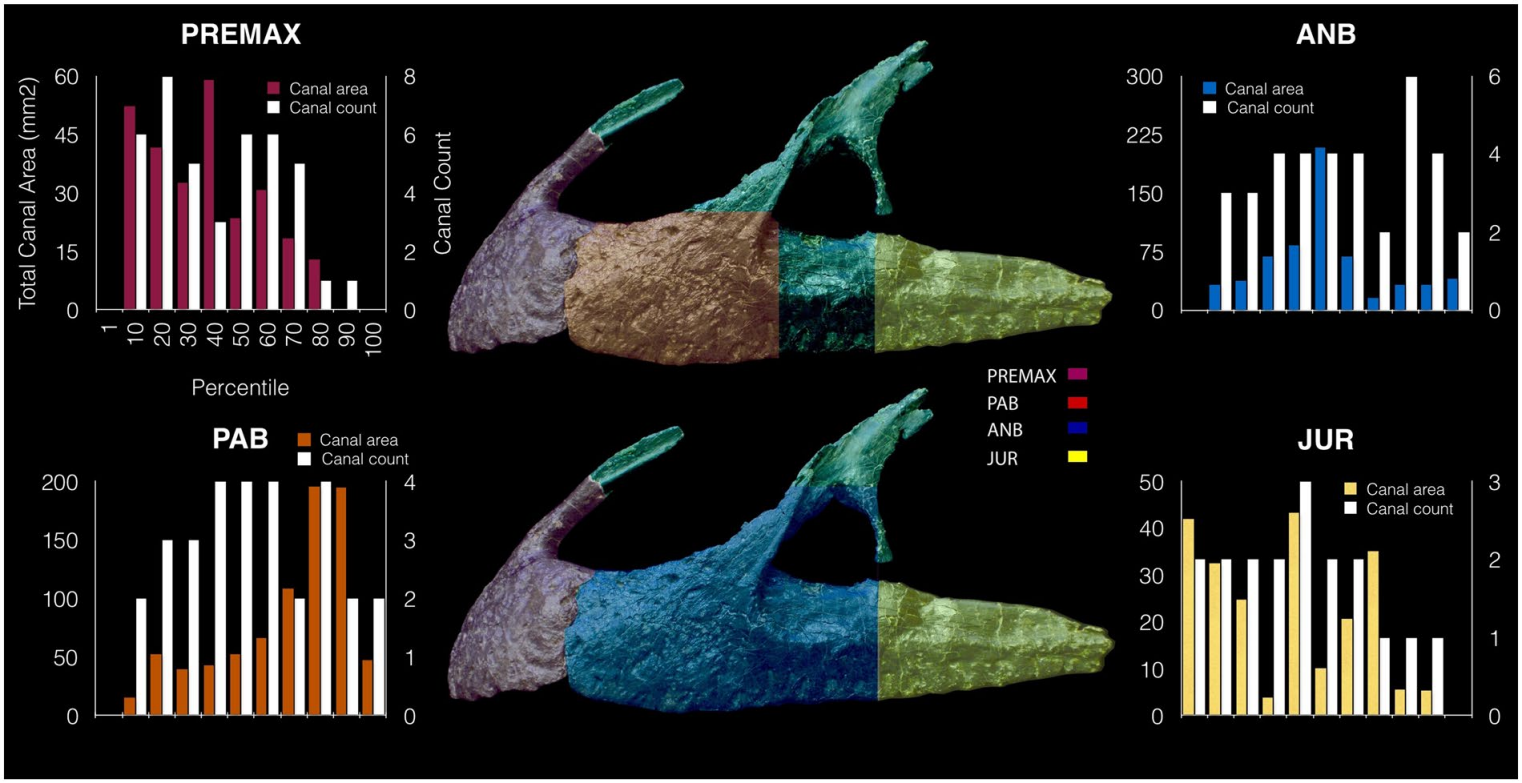
Lateral view of left premaxilla and maxilla, denoting maxillary anatomy as suggested by Hendrickx and Mateus^56^. Graphs denote changes in canal area versus canal count (i.e. number of observable canals per slice) at various regions of premaxillary and maxillary anatomy, based on data from Supplementary Table S1. Abbreviations: **premax**: premaxilla; **pab**: preantorbital body; **anb**: anterior body; **jur**: jugal ramus.

We interpret all of these branching structures as part of the neurovascular system, independent of and separate from the pneumatic system. Branching is most complex in the premaxilla where much of the premaxillary body is occupied by canals of a complex, dendritic or anastomosing form (Figs 1 and 3B). One premaxillary canal appears to ascend dorsally through at least the base of the nasal process of the premaxilla, although its dorsal extent is unclear. Much of the length of the maxilla is occupied by a large, serpentine canal that extends through the maxillary body at mid-height (Figs 2B and 3B). At a point ventral to the base of the ascending ramus, the canal is more dorsally positioned and possesses a ganglion-like thickening that sends off dendritic branches dorsally, ventrally, and anteriorly. This thickening is denoted in Fig. 4 as the elevated plateau within the area data for the preantorbital body, coupled with a decrease in dendricity. It is associated with the presence of especially large foramina near the base of the ascending ramus, while ventral branches pass dorsal and lateral to the alveoli. The majority of the foramina associated with this maxillary branch are found on the preantorbital body, forming medial maxillary foramina, mirroring the most dendritic sections of the maxillary neurovascular canals (Figs 2B, 3B and 4). This is reflected by the increased canal:bone ratio seen in Table 1 compared to the anterior body as a whole. The remainder of the foramina are found ventrally along the tooth row (maxillary alveolar foramina), as well as along the antorbital ridge (maxillary circumfenestra foramina) (Fig. 2A). Interestingly, the latter two foramina types appear to have very little interaction with the maxillary branch of the canals (Figs 2 and 3B), and these various canals and their sub-branches are highly variable in size. Taken together, these structures occupy *at least* 7.3% and 6.7% of the internal volume of the premaxilla and maxilla, respectively (Table 1), and an accessory sinus is linked to the premaxillary fenestra at the base of the ascending ramus of the maxilla. Several branches of these maxillary neurovascular structures closely approach the sinus although they do not appear to interact with it directly.

## Discussion

The presence of foramina on the lateral surfaces of the premaxilla and maxilla are typical, widespread amniote features^13^ and only rarely are they given more than fleeting mention in descriptions and analyses. Nevertheless, a long-standing debate concerns the size, number, and distribution of these foramina in non-avialan dinosaurs as well as the presence of either keratinous rhamphotheca or lip-like and/or cheek-like extraoral tissues^14–22^. It is currently thought that keratinous rhamphotheca were present in ornithischians and in such theropods as ornithomimosaurs, therizinosaurs, oviraptorosaurs and some ceratosaurs; the condition in other lineages of non-avialan theropods remains ambiguous but the absence in *Neovenator* of osteological correlates linked to the presence of rhamphotheca, including bone spicules and shallow, obliquely oriented foramina^23^, indicates that such tissues were absent. Typically, fewer and larger foramina are seen in organisms with extensive extraoral tissues (e.g. lips, 13), and the presence of a large number of foramina on the premaxilla and maxilla in *Neovenator* suggests that whatever extraoral tissue was present was immobile. The rugosity of bones in this region is comparable to that of abelisaurids and carcharodontosaurids^2^, although *Neovenator* is somewhat atypical regarding foramina count being most comparable to abelisaurids such as *Majungasaurus* ^24^ where sculpted cranial morphology and foramina count have been linked with dermal specialisations. It is important to note that the presence of cornified, specialised dermal tissue is not itself incompatible with the presence of enhanced sensory capabilities; the crocodylian face is covered by an extensively cracked epidermis, nearly twice as thick as anywhere else on the body^25^, yet these archosaurs are capable of extraordinary facial sensitivity. Furthermore, the presence of a sensitive scaly integument has been suggested for the tyrannosaurid *Daspletosaurus horneri* ^11^.

**Figure 5:**
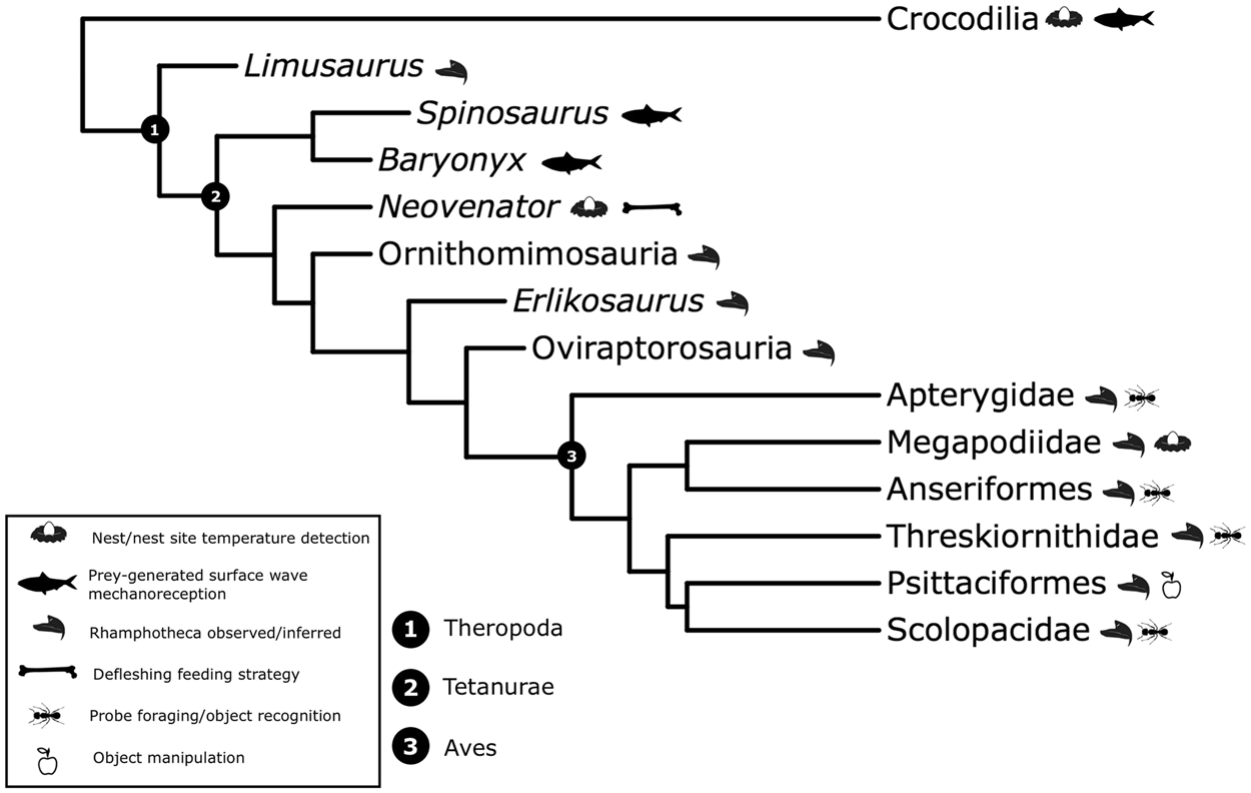
A schematic phylogeny representing the archosaurs mentioned in the text, and their inferred palaeoecology as a result of the presence of mandibular foramina an/or internal neurovascular structures.

In modern archosaurs, the function of foramina is easier to ascertain as they are often associated with mechanoreceptors (Fig. 5). In the rostrum of birds for example, Grandry and Herbst corpuscles give probe foragers (i.e., Apterygidae, Scolopacidae, and Threskiornithidae) the tactile ability to recognise prey items during foraging^26^. In Anseriformes, neurovascular foramina on the tips and ridges of the beak allow for the detection, recognition, and transport of food^27^, while enhanced object manipulation is seen in Psittaciformes^28^. Several ratite species also show advanced bill sensitivity, however the extent of this is yet to be tested histologically^29^. In crocodylians, aforementioned mechanoreception is similarly present in the rostrum, with neurovascular foramina representing an exit for the highly sensitive branches of the trigeminal nerve (CN V)^30^. The existence of substantial innervation within the rostrum, associated with foramina, has also been associated with hunting and foraging in several other Mesozoic fossil reptiles^10–12^. A similar structure is seen in extant varanids^30, 31^, however, it is less dendritic than is the case in *Neovenator*, and squamate’s premaxillae lack the foramina seen in archosaurs. Varanids were thus not considered further for the purpose of this study.

We suggest that the structures we have described in *Neovenator* represent branches of the trigeminal nerve similar to those seen in extant crocodylians^30^. These neurovascular canals can be divided into two branches; in the premaxilla, they form part of the ophthalmic (CN V_1_) division of the trigeminal nerve, specifically, the premaxillary branch of its medial nasal ramus (Fig. 1)^24^. This is the smallest of the three divisions and has a purely sensory function^32^. The maxillary (CN V_2_) branch occupies the maxilla, relaying information from the upper jaw and teeth (Fig. 2)^9, 32^. Our identification of this structure in *Neovenator* as the ophthalmic and maxillary branches of the trigeminal nerve is consistent with its termination at numerous neurovascular foramina that pepper the bones of the skull (Figs 1A and 2A)^9, 12, 13^.

A portion of the subnarial artery and vein, as well as maxillary vessels, are most likely also present in *Neovenator* (Fig. 2B), with the latter merging with the nerve to form the maxillary canal^33^. However, it should be noted that making comparisons with the cranial vasculature of extant archosaurs is hindered by the highly modified nature of their skulls relative to those of Mesozoic theropods; for example, the crocodylian skull is platyrostral as opposed to oreinirostral, the antorbital fenestra is absent, and a secondary palate is present^34^. In addition, it is difficult to discern differences between neural and vascular tissue in fossils due to issues of preservation. Furthermore, the large size discrepancy present between extinct dinosaurs like *Neovenator* and extant taxa increases the difficulty in assigning functions to extinct taxa based on osteological structures present in the latter, arguably requiring “extrapolation beyond empirical data”^35^.

The extensive cephalic vasculature of crocodylians has a thermoregulatory function, as both the oral and nasal regions act as major cranial sites of thermal exchange, with the latter particularly vascularised^34^. Similarly, many extant birds employ cephalic vasculature for thermoregulatory means^36^. In both of these extant archosaur groups, a highly vascularised oral region can form a palatal plexus, with arterial and venous branches of the palatine blood vessels anastomosing to form a large surface area for thermal exchange^34, 36^. The highly vascularised nasal regions in birds have also been shown to be areas of thermal exchange^36–38^; a similar pattern is present in crocodylians where a thermoregulatory function has yet to be rigorously tested^34^. It is possible that a similar function was present in *Neovenator*. However, although the narrow rostrum would have reduced the surface area for palatal heat exchange, anteroventral distribution of the canals and their associated foramina is surprising within the context of a thermoregulatory function as this position would not make use of the entirety of the lateral surfaces of these two bones. Furthermore, there is currently no evidence for a plexus-type complex in *Neovenator*. For these reasons we consider it unlikely that temperature control was an important function of these neurovascular structures.

Returning to our trigeminal nerve hypothesis, innervation linked to external integumentary sensory organs (ISO) in extant crocodylians allows the snout to perform as a specialised tactile organ that perhaps possesses ‘sensitivity greater than primate fingertips’^39^. Tests on juvenile alligators have shown how ISOs aid mechanoreception, the detection of prey-generated ripples, accurate biting, and ‘tactile discrimination of items held within the jaws’^39^. Erickson *et al*.^40^ also regarded ISOs as a tool to determine the application of bite force when feeding. However, mechanoreception is not the only function of ISOs in crocodylians; these organs also have a combined thermo- and chemosensory role as well as responding to pressure^41^.

Within some Mesozoic theropods, the presence of enlarged foramina on the rostrum as well as a large neurovascular cavity in *Spinosaurus* (and potentially *Baryonyx* ^12^), documented via μCT-scanning, have been linked to a specialised nervous system and correlated with a semi-aquatic lifestyle (Fig. 5)^10, 12^. However, despite the significant number of theropod skulls that have now been subjected to μCT scanning, neurovascular anatomy linked with exceptional tactile ability has only been described in spinosaurids. The new data presented here, coupled with the recent finding in tyrannosaurids^11^, indicate that spinosaurids were not exceptional within Theropoda in terms of their facial neuroanatomy, and that *Neovenator*, apparently a ‘typical’ terrestrial theropod lacking any specialisations for amphibious or aquatic life, possessed a comparable degree of neuroanatomy and presumed rostral/facial sensitivity. Although it remains possible that *Neovenator* was a specialized aquatic forager and that this unusual configuration can be linked to such a lifestyle, this inference is not reflected in any other aspect of *Neovenator*’s anatomy, or from the palaeoecological and palaeoenvironmental context in which *Neovenator*, other neovenatorids, carcharodontosaurians, and allosauroids are preserved. While opportunistic foraging of aquatic prey remains possible for *Neovenator*, combined evidence contradicts a fundamental reinterpretation of this animal’s palaeoecology.

What appears much more parsimonious is that a previously under-appreciated degree of rostral and facial sensitivity was typical for large tetanurans, and that this played a role in how and where bites were applied, as well as in other aspects of prey-handling and behaviour. Two-dimensional analysis of dental microwear (Barker *et al*. in preparation) suggests that *Neovenator* actively avoided tooth-bone contact; two maxillary teeth bear scratch-dominated enamel with minimal pitting, a configuration characteristics of extant carnivores that avoid bone contact (e.g. cheetahs)^42, 43^. We therefore speculate that a sensitive snout would be useful when defleshing a carcass, imparting enhanced sensitivity to allow the animal to carefully differentiate between meat and bone when feeding. In addition, this ability would help explain the rarity of evidence for tooth-bone contact in the non-tyrannosaurid Mesozoic fossil record^9, 44^. We also note that enhanced facial sensitivity may potentially have played a role in intraspecific communication, consistent with data suggesting that ritualised face-biting – and thus facial contact in general – was part of theropod behavioural repertoire^11, 45^. Coronal CT slices are also known for a ventrally compressed *Tyrannosaurus rex* ^46^ specimen and reveal a (presumably deformed) maxillary channel and nerve (V_2_). Tyrannosaurids show evidence of cranio-facial biting, this presumably reflecting intraspecific combat behaviour^45, 47–49^. It would be of interest to compare the size of the neurovasculature in tyrannosaurids to the other theropods discussed here (e.g. *Neovenator*) and assess whether this is simply a retained plesiomorphic trait or was reduced, perhaps as an evolutionary response either to this aggressive behaviour or to the unusual feeding strategy (involving increased tooth-bone contact) of this clade. A thermosensory role also remains possible, responses to thermal stimuli potentially proving useful within the context of finding and maintaining a nest environment by analogy with behaviour present in crocodylians^50–53^ as well as megapode birds^54^. It is feasible to envisage *Neovenator* employing its snout whilst engaging in nesting behaviour, especially given that crocodylians and most non-avian theropods appear to share mound building behaviour^55^.

In conclusion, the identification of a complex and highly developed neurovascular network in the rostrum of *Neovenator*, an otherwise ‘typical’ allosauroid theropod, places previous inferences of such structures in spinosaurids in a new context. While it is possible that spinosaurids secondarily adapted the neuroanatomy described here for aquatic foraging^56^, it is also possible that the relevance of facial sensitivity to this behaviour has been over-stated. Further analyses on the morphology and distribution of cranial nerves within Dinosauria, using their extant archosaur cousins as reference, is required in order to better understand and quantitatively assess the use of facial sensitivity in these animals, a concept supported by foramina studies in extant ratites^29^. By standardising our results using percentiles across key anatomical references, our data can be used to facilitate such further analysis.

## Material and Methods

Both left and right premaxillae, the left maxilla, and the right nasal from the *Neovenator* holotype (MIWG 6348) were scanned at the μ-VIS X-Ray Imaging Centre at the University of Southampton (UK), using the custom designed Nikon/Metris dual source high energy micro-focus walk-in enclosure system. A 450 kVp source was used, coupled with a 1621 PerkinElmer cesium-iodide detector. Peak voltage was set at 400 kVp and the current at 314 μA (125.6 W). A total of 3142 projections (16 frames per projection) were collected during a 360° rotation, with each projection occurring over an exposure time of 177 ms. The raw projection data were reconstructed into 3D volumes (32-bit raw) using isotropic voxels (voxel dimension = 124.7 µm^3^) by means of Nikon’s reconstruction software (CT Pro 3D, v. XT 2.2 SP10), which uses a filtered back projection algorithm.

Subsequently the reconstructed 32-bit volumes were downsampled to 8-bit (raw) volume files to reduce processing power and computational load of the processing workstations. The 8-bit volumes were then imported to VG Studio Max (version 2.1 Volume Graphics GmbH, Heidelberg, Germany) for further processing and visualisation. Segmentation in Avizo® was predominantly performed manually on a slice-by-slice basis using the “Brush” tool, although semi-automatic segmentation was possible on certain elements using the “Magic wand” tool, at grey-scale values of 197 with the tolerance set at 45. Due to resolution limitations, we focused our quantitative analyses on the lower boundary estimate of the structure and volume of the neurovascular canals. Our results and discussion are mainly based on the left premaxilla and maxilla, where the relevant internal structures are best preserved (Figs 1, 2 and 3). Figures were compiled and annotated in Adobe Illustrator (Adobe Systems Inc., San Jose, California), using terminology based on *Torvosaurus* material^57^. Percentiles (Supplementary Table S1) were generated using the scan slice numbers in order to standardise our results.

## Electronic supplementary material

Supplementary Table S1
