# Supplementary material for: Complex neuroanatomy in the rostrum of the Isle of Wight theropod Neovenator salerii
Source: Sci Rep. 2017 Jun 16;7:3749. doi: 10.1038/s41598-017-03671-3 (PMC5473926; doi:10.1038/s41598-017-03671-3)
Supplement: Supplementary file 1 — Supplementary Table S1 [file 41598_2017_3671_MOESM1_ESM.pdf]

Complex neuroanatomy in the rostrum of the Isle of Wight theropod *Neovenator salerii*

Chris Tijani Barker[1], Darren Naish[1], Elis Newham[2], Orestis L. Katsamenis[3] and Gareth Dyke[4]

[1] Ocean and Earth Science, National Oceanography Centre, University of Southampton, European Way, Southampton SO13 3ZH, UK

[2]Faculty of Engineering and the Environment, University of Southampton, SO17 1BJ, Southampton, UK

[3] μVIS X-ray Imaging Centre, Faculty of Engineering and the Environment, University of Southampton, SO17 1BJ, Southampton, UK

[4] Department of Evolutionary Zoology and Human Biology, University of Debrecen, 4032 Debrecen, Egyetem tér 1, Hungary

Supplementary Table S1. Neurovascular canal area, count (i.e., dendricity) and average area per canal in percentiles (slices) across several areas of *Neovenator*'s rostral anatomy. Percentiles were generated using slice numbers for that particular anatomical feature. Abbreviations: **premax**: premaxilla; **pab**: preantorbital body; **anb**: anterior body; **jur**: jugal ramus.

Region	Percentile	Slice №	Nerve Area (mm²)	Nerve Count	Area/Count
PREMAX	1	215	0	0	0
	10	250	52.40	6	8.73
	20	285	41.63	8	5.20
	30	320	32.63	5	6.53
	40	355	59.03	3	19.68
	50	390	23.67	6	3.95
	60	425	30.88	6	5.15
	70	460	18.33	5	3.67
	80	495	13.02	1	13.02
	90	530	0.28	1	0.28
	100	560	0	0	0
PAB	1	173	0	0	0
	10	264	15.13	2	7.56
	20	355	52.50	3	17.50
	30	446	38.98	3	12.99
	40	537	42.55	4	10.64
	50	628	52.42	4	13.11
	60	719	66.44	4	16.61
	70	810	108.14	2	54.07
	80	901	195.11	4	48.78
	90	992	194.86	2	97.43
	100	1083	46.86	2	23.43
ANB	1	173	0	0	0
	10	319	31.83	3	10.61
	20	465	38.03	3	12.68
	30	611	68.25	4	17.06
	40	757	82.83	4	20.71
	50	903	207.78	4	51.95
	60	1049	68.08	4	17.02
	70	1195	15.19	2	7.59
	80	1341	32.23	6	5.37
	90	1487	31.94	4	7.98
	100	1636	40.28	2	20.14
JUR	1	1637	41.97	2	20.98
	10	1729	32.59	2	16.30
	20	1821	24.86	2	12.43
	30	1913	3.88	2	1.94
	40	2005	43.38	3	14.46
	50	2097	10.03	2	5.02
	60	2189	20.72	2	10.36
	70	2281	35.09	1	35.09
	80	2373	5.69	1	5.69
	90	2467	5.41	1	5.41
	100	2556	0	0	0
